# Discordant Eosinophilic/T-Cell Chorionic Vasculitis in a Dichorionic Diamniotic Placenta

**DOI:** 10.3390/ijms24119207

**Published:** 2023-05-24

**Authors:** Evelina Silvestri, Francesca Servadei, Ione Tamagnini, Laura Moretti, Maria Paola Bonasoni

**Affiliations:** 1 Unit of Fetal and Neonatal Pathology, Division of Pathology, San Camillo-Forlanini Hospital, 00152 Rome, Italy; esilvestri@scamilloforlanini.rm.it; 2 Anatomic Pathology, Department of Experimental Medicine, University of Rome “Tor Vergata”, 00133 Rome, Italy; francescaservadei@gmail.com; 3 Pathology Unit, Azienda USL-IRCCS di Reggio Emilia, 42123 Reggio Emilia, Italy; ione.tamagnini@ausl.re.it (I.T.); laura.moretti@ausl.re.it (L.M.)

**Keywords:** Eosinophilic/T-Cell chorionic vasculitis, twin placenta, discordance, TIA-1 concordance

## Abstract

Eosinophilic/T-cell chorionic vasculitis (ETCV) is an idiopathic lesion composed of eosinophils, CD3+ T lymphocytes, and histiocytes. In twins, ETCV may affect only one chorionic plate, a feature defined as “discordant”. We present a case of ETCV discordance in a diamniotic dichorionic placenta at 38 weeks of gestation, in which the female twin was small for gestational age, weighing 2670 g (25th percentile). The corresponding placental territory presented ETCV in two close chorionic vessels with concordance of the fetal inflammatory response. Immunohistochemistry showed an abundance of CD3+/CD4+/CD25+T lymphocytes, CD68 PG M1+ macrophages, and scattered CD8+ T cells with focal TIA-1 positivity. Granzyme B, CD20 B lymphocytes, and CD56 natural killer cells were negative. High-grade villitis of unknown etiology (VUE) was additionally found and displayed comparable ETCV findings, except for an equivalent ratio of CD4+/CD8+ T cells, but TIA-1 was focally expressed. VUE was associated with chronic histiocytic intervillositis (CHI). The combination of ETCV, VUE, and CHI may have been responsible for reduced fetal growth. Concordance was observed in the ETCV and TIA-1 expression, both in ETCV and in VUE, which is a maternal response. These findings may suggest a common antigen or chemokine pathway to which both mother and fetus accordingly responded.

## 1. Introduction

Eosinophilic/T-cell chorionic vasculitis (ETCV) is an unusual idiopathic lesion in which the chorionic vasculitis is mainly composed of eosinophils and CD3+ T lymphocytes. The first description was reported in 2002 by Fraser and Wright [1], who defined it as “a new form of chorionic vasculitis characterized by an infiltrate composed primarily of eosinophils and CD3+ T lymphocytes that very focally involve a single chorionic vessel (artery or vein), which radiates away from the amniotic fluid (i.e., toward the intervillous spaces), and that may extend into stem villous vasculature; this lesion occurs in the absence of any evidence of chorioamnionitis” [1].

Since then, ETCV has been further characterized and can affect multiple chorionic vessels, even extending to stem vessels. The inflammatory infiltrate may be oriented toward the intervillous space, the amniotic fluid, both or ambiguous; in association, acute chorioamnionitis and chronic villitis have been described [2,3,4,5,6,7].

According to the literature, ETCV has been reported in only 16 twin placentas and 1 triplet placenta [1,3,6,7]. In twins, despite chorionicity, ETCV may affect only one chorionic plate, and this difference has been defined as “discordance” or “discordant”, as recently investigated by Nohr and Wright [7]. In this study, the histological features of ETCV were different between the twins in terms of severity of inflammation, number of vessels involved, extension, and twin growth. ETCV was present in isolated chorionic vessels, mostly situated close to the cord insertion, and the inflammatory response was segmental in the vessel wall. These findings may correlate with unexpected growth restriction or small for gestational age (SGA) babies, from both twin and singleton placentas. Although the CD3+ T response, including T-regulatory cells (Tregs), has been well documented, and in-depth characterization of the different subsets of lymphocytes is still lacking [3,6].

In this paper, we present a case of ETCV discordance in a diamniotic dichorionic (Di-Di) placenta, in which the twin affected was also SGA. The ETCV histological findings, location, and inflammatory response are described and discussed. Immunohistochemistry was performed to investigate the inflammatory infiltrate, with further characterization of the different classes of lymphocytes, highlighting the positivity for TIA-1, a constituent of cytotoxic T lymphocyte granules involved in cell apoptosis.

## 2. Case Description

A 30-year-old woman had periodical ultrasound scans at our institution for a dichorionic diamniotic twin gestation. Maternal clinical history was uneventful. At 38 weeks, preterm labor occurred, and an urgent Caesarean section was performed. At birth, the male twin weighed 3600 g (90th percentile for gestational age), and the female weighed 2670 g (25th percentile), consistent with SGA [8]. The Di-Di placenta was examined and sampled according to the Amsterdam criteria [9], and the findings showed fused chorionic discs and common membranes, which divided the siblings’ territories, and the male part was identified by a clip clamped on the umbilical cord.

This part weighed 387 g and was sized 14 × 17 cm, with a thickness between 2 cm and 3 cm, the latter measurement corresponding to a small succenturiate lobe. The cord was 38 cm in length, presented three vessels, and inserted at 2 cm from the edge, close to the common membranes. The fetal surface showed multiple small subchorionic fibrinoid plaques. The maternal surface was incomplete for about 10% of the surface due to absent decidua. Histology showed non-occluding recent thrombi in the vessels at cord insertion and in the chorionic plate. The parenchyma showed regular maturation for gestational age.

**Figure 1:**
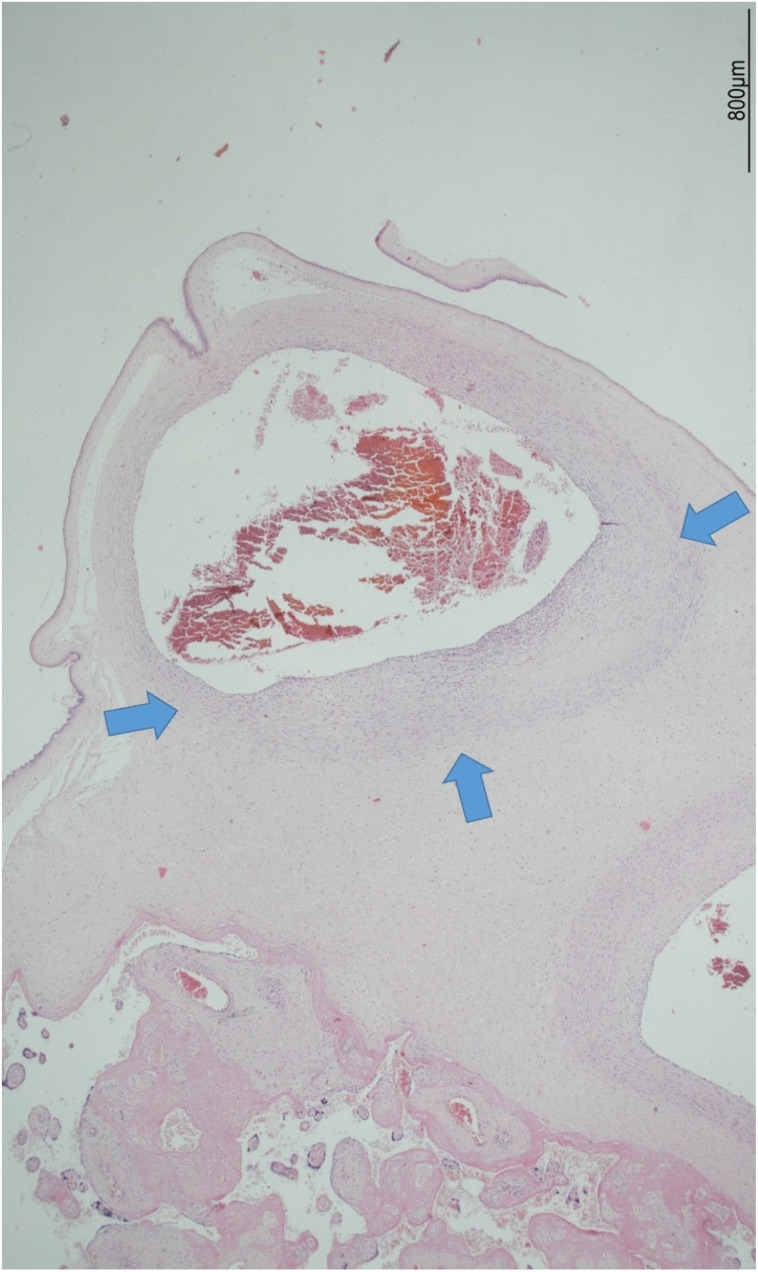
ETCV in a chorionic vessel: the inflammatory response affected the lower half of the wall (the blue arrows highlight the involvement of the vessel). The inflammatory cells always faced the villi (hematoxylin and eosin, 2HPF).

**Figure 2:**
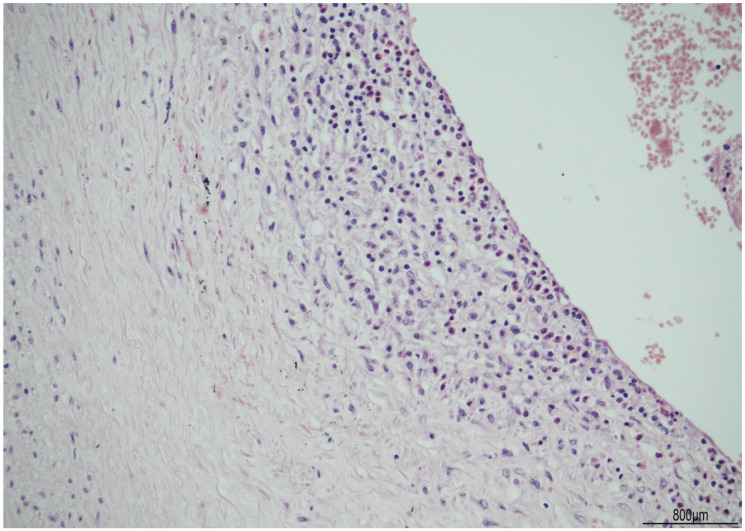
ETCV inflammatory infiltrate: the cell-mediated immune response within the vascular wall was composed of lymphocytes, macrophages, and scattered eosinophils.

**Figure 3:**
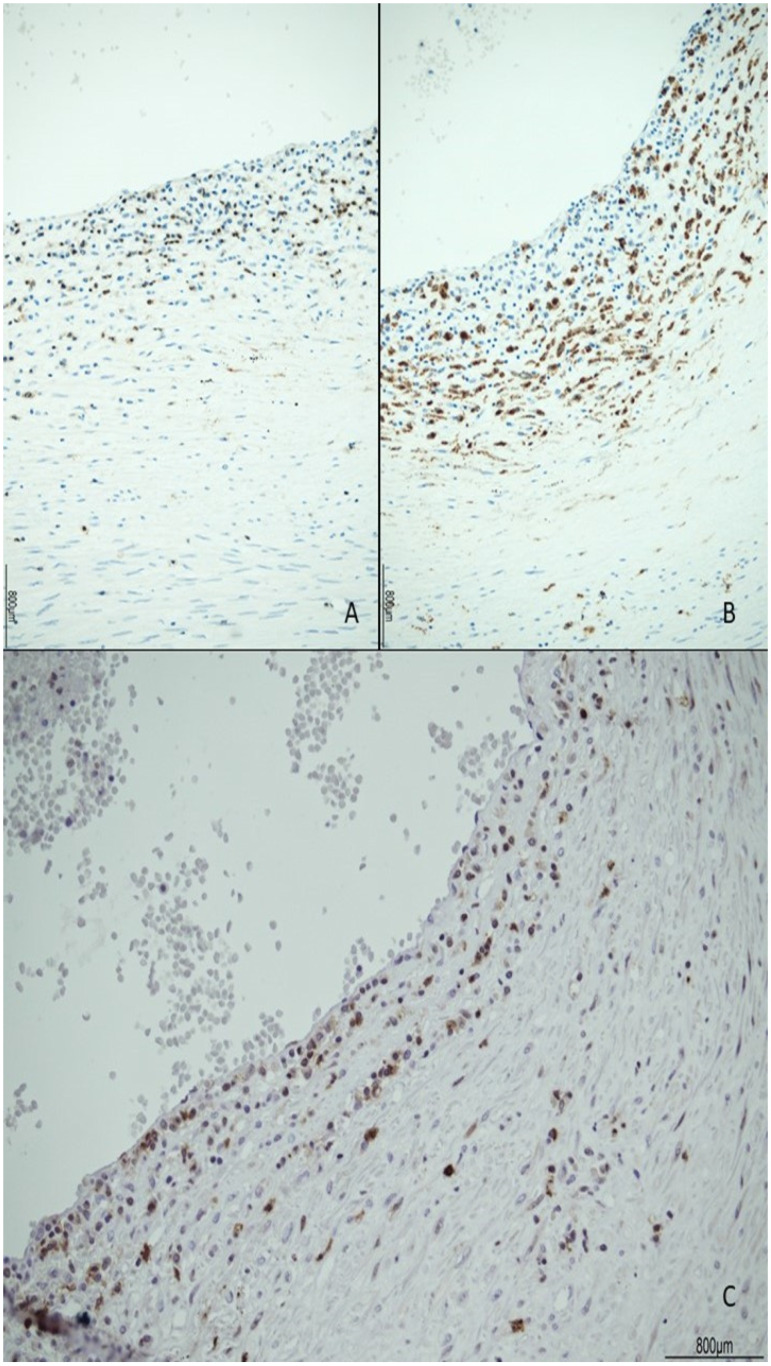
ETCV immunophenotype: the inflammatory infiltrate was made of abundant CD3+ T lymphocytes (**A**) and CD68 PGM1+ macrophages (**B**). CD25+ T regulatory cells were also numerous (**C**).

**Figure 4:**
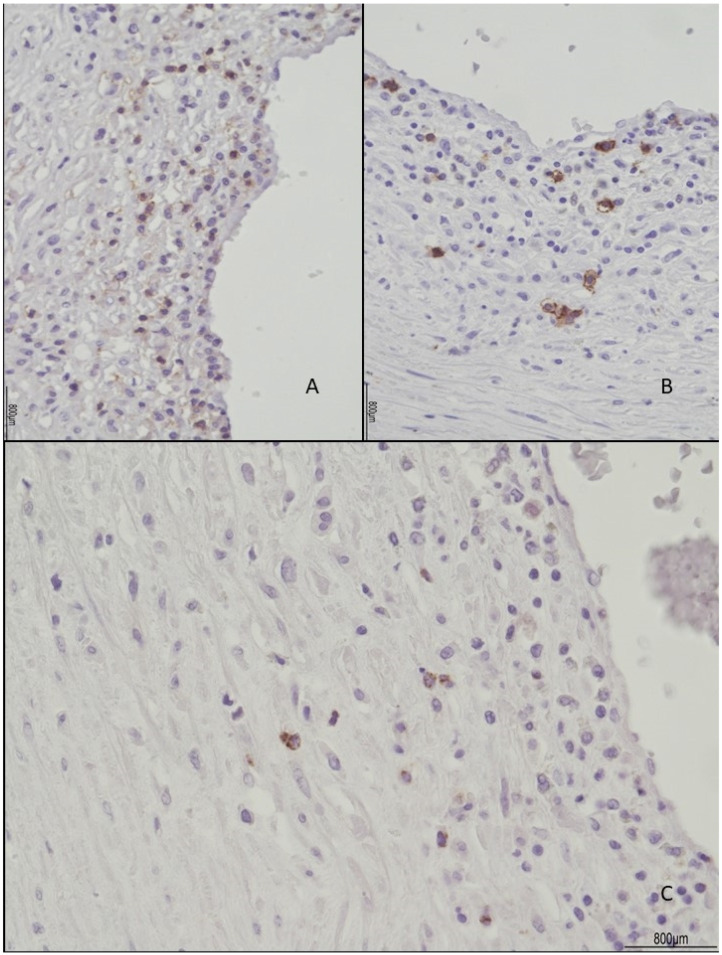
Lymphocyte immunophenotype: CD4+ T cells (**A**) outnumbered CD8+ T cytotoxic cells (**B**), which focally expressed TIA-1 (**C**). The ratio between CD4+ and CD8+ T cells was estimated in a semiquantitative way by counting them in three fields at 20 HPF.

The female twin territory weighed 344 g and was sized 16 × 13 cm, with a thickness between 3 cm and 1 cm, and the latter extended for 40% of the volume. The three-vessel umbilical cord was 30 cm in length and inserted at 4 cm from the chorionic edge. The fetal and maternal surfaces were unremarkable. Histological examination revealed ETCV in two close chorionic vessels (distanced by 6 mm). In one of them, the vasculitis involved almost half of its circumference and always faced the villi (Figure 1). In the other, the inflammatory cells were circumferentially situated and faced both the amniotic cavity and the parenchyma. In both vessels, ETCV was represented by a cell-mediated immune response composed of lymphocytes, histiocytes, and eosinophils (Figure 2). Immunohistochemistry displayed numerous CD3+/CD25+ T lymphocytes and CD68 PGM1+ macrophages. CD4+ T helper cells outnumbered CD8+ T cytotoxic cells (Figure 3 and Figure 4). The ratio was estimated in a semiquantitative way by counting them in three fields at 20 HPF. A few cytotoxic lymphocytes focally expressed TIA-1 (Figure 4C) but were negative for granzyme B. Immunohistochemistry for CD20 B lymphocytes and CD56 natural killer cells was negative.

**Figure 5:**
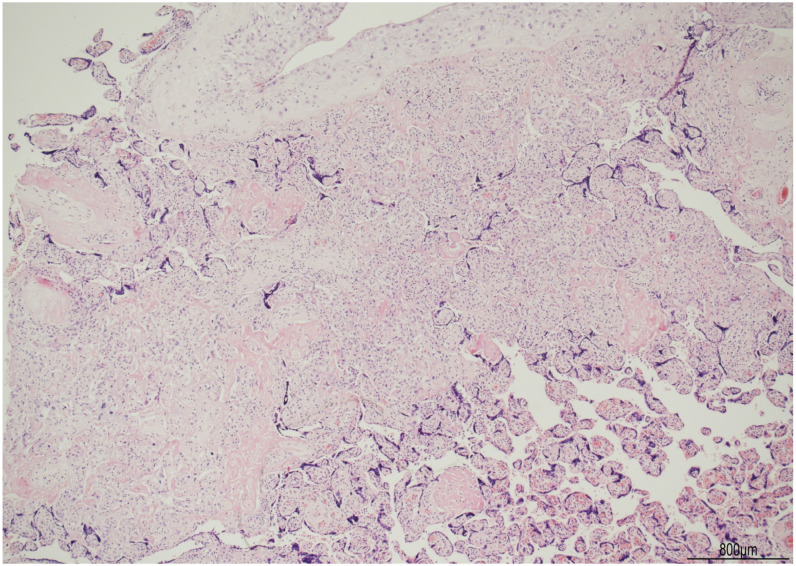
High-grade VUE: this lesion was classified as such because more than 10 contiguous villi were affected by chronic villitis in a single focus (hematoxylin and eosin, 4HPF).

**Figure 6:**
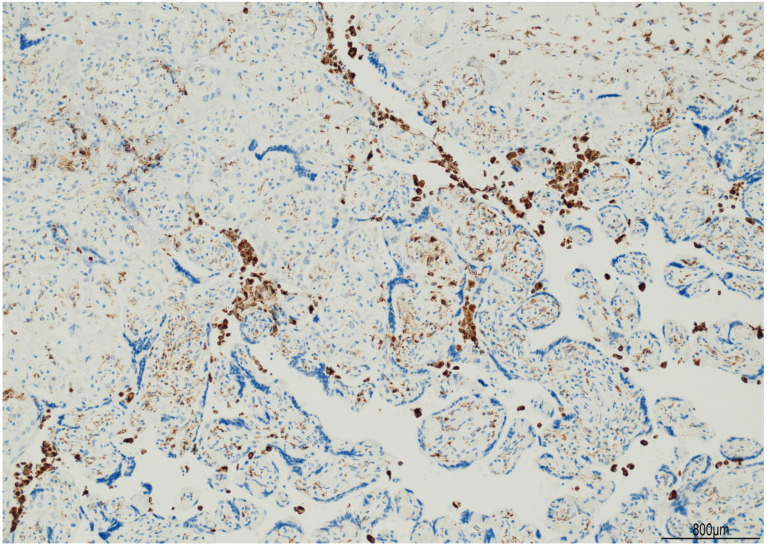
Chronic histiocytic intervillositis: in the area of high-grade VUE, CD68 PGM1+ macrophages were scattered in the intervillous space.

**Figure 7:**
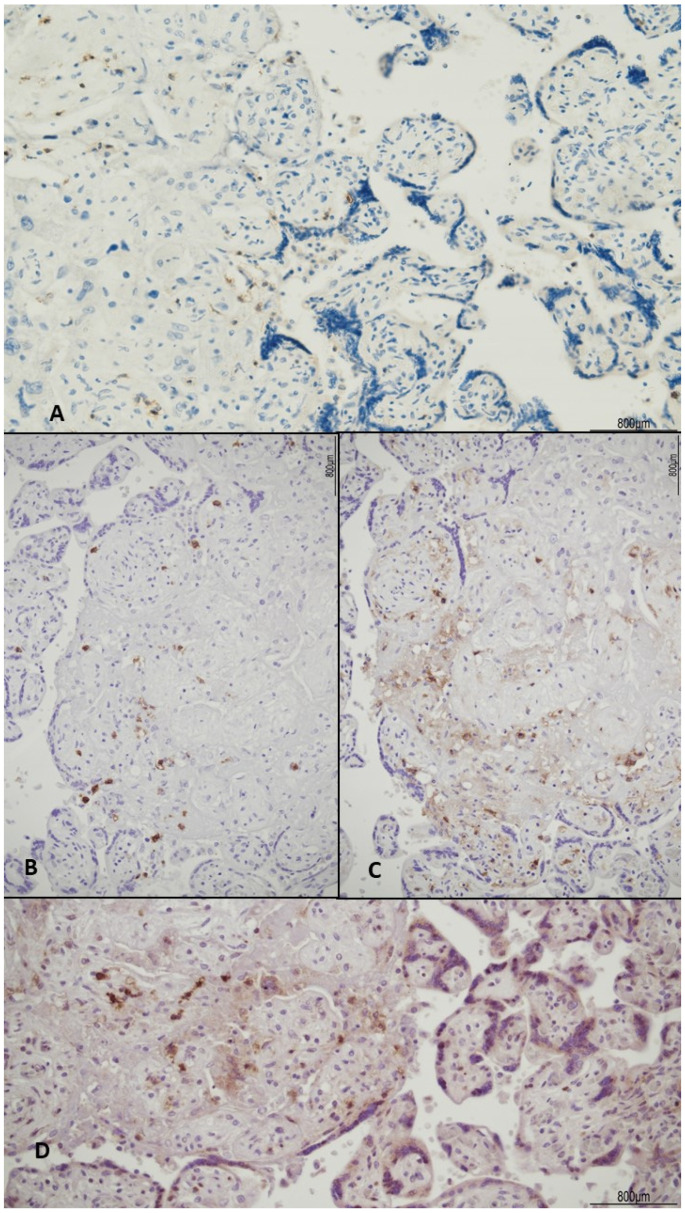
High-grade chronic villitis: CD3+ T lymphocytes were observed in the villous stroma (**A**), CD4+ (**B**) and CD8+ cells (**C**) presented a similar ratio (CD4+/CD8+), evaluated in a semiquantitative way by counting them in three fields at 20 HPF. Scattered CD25+ T regulatory cells (**D**) were detected.

**Figure 8:**
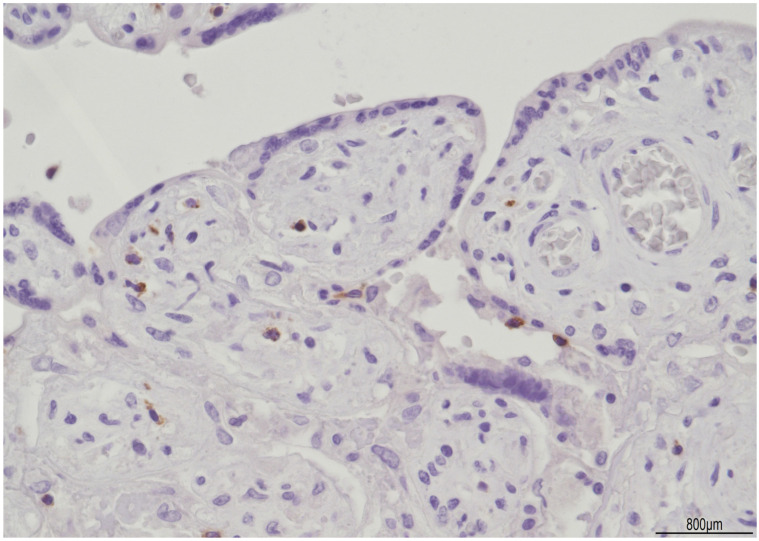
High-grade chronic villitis: TIA-1 was focally expressed by cytotoxic lymphocytes.

In the parenchyma, patchy chronic villitis of unknown etiology (VUE) was observed and classified as high-grade, according to the Amsterdam criteria [9], as more than ten contiguous villi were affected, in at least one focus (Figure 5). High-grade VUE was associated with chronic histiocytic intervillositis (CHI), as evidenced by CD68 PGM1+ macrophages (Figure 6). Further immunohistochemical characterization of VUE showed CD3+ T lymphocytes with CD4+ and CD8+ cells in a similar ratio and detection of CD25+ T cells (Figure 7). TIA-1 was focally expressed by cytotoxic cells (Figure 8), but granzyme B was not observed. CD20 B lymphocytes and CD56 natural killer cells were not detected.

The antibodies employed to evaluate ETCV and VUE are shown in Table 1.

## 3. Discussion

ETCV is an unusual chronic inflammatory lesion with an estimated incidence of 0.2 to 0.7% in pregnancy [10,11]. Nohr and Wright reported an ETCV incidence of 0.6% in fused twin placentas over a 13-month period (multifocal ETCV found in six multiple gestations out of 950 samples, singletons included) [7]. As a mostly focal lesion, the exact incidence of ETCV is difficult to ascertain, being underestimated by standard placental sampling [3]. Moreover, the inflammatory infiltrate is detected in multiple chorionic vessels and extends to the stem vasculature, with various patterns of orientation (towards the villi, the amniotic cavity, both or ambiguous); acute chorioamnionitis and VUE may also be associated [3,4,5]. In the past, ETCV had been reported as “discordant” in twin and triplet placentas [1,3,6,7], as the inflammatory infiltrate was variably present in the siblings’ placental territories, but Nohr and Wright tried to better analyze this phenomenon [7]. They described six twin placentas, two diamniotic monochorionic (Di-Mo), and four Di-Di. In five out of six, ETCV was multifocal and discordant, but, in one Di-Di (case 3), there was twin concordance, with ETCV in both territories. However, in the region with more extensive ETCV, VUE was also found, and the twin presented intrauterine growth restriction (IUGR). In another Di-Di (case 6), ETCV and VUE coexisted. The clinical implications of ETCV, such as IUGR, remain uncertain but may be due to fetal vascular malperfusion induced by endothelial injury or altered blood flow [7].

In the Di-Di placenta that we described here, ETCV was observed in only one territory, in which the baby was SGA. The inflammatory infiltrate was multifocal and involved two close vessels (distanced by 6 mm). In one chorionic vessel, the inflammatory infiltrate was found in half of the wall, facing the villi, but, in the other one, it was circumferential. As previously observed [3,7], the inflammatory infiltrate orientation did not seem significant, as various patterns of distribution were described: towards the amniotic cavity, the parenchyma, or not definable.

Regarding the composition of the inflammatory infiltrate, in ETCV, it is typically composed of lymphocytes, CD68+ macrophages, and eosinophils. Immunophenotyping revealed the presence of CD4+ and CD8+ T lymphocytes and FOXP3+ and CD25+ Tregs, whereas CD56+ natural killer cells were not detected [3,6], and CD20+ B cells were inconsistently present [3]. ETCV is interpreted as a T helper type 2 immune response (TH2) due to the simultaneous presence of T cells and eosinophils [12]. Moreover, it is a fetal immune response, similar to acute chorionic vasculitis, as proved by fluorescent in situ hybridization (FISH). In 31 placentas, FISH was carried out on 400 interphase nuclei at the location of ETCV to analyze the number of XX, XY, X, and Y cells by fluorescent X- and Y-chromosome probes. In 6/31 placentas, in which the newborn was male, the average percentage of XY nuclei was between 70 and 90, confirming the fetal origin of the inflammatory cells [13].

CD4 and CD25 are markers of Tregs; in particular, CD25 is part of the IL-2 receptor and regulates cell proliferation, apoptosis, Tregs, and T effector (Teffs) lymphocytes. Tregs are fundamental in maintaining self-tolerance, and their absence or abnormal expression can lead to autoimmune diseases [14,15]. CD25 is also expressed by some subsets of CD8+ T lymphocytes, which are also classified within the Tregs population [16]. In ETCV, it has been suggested that Tregs modulate inflammation, limiting autoimmunity damage, or may be a reaction towards an unknown infective agent [6].

In our case, the ETCV inflammatory infiltrate was composed of CD3+ T lymphocytes, with a prevalence of CD4+/CD25+ T subsets, scattered CD8+ T cytotoxic cells with focal TIA-1 positivity, CD68 PGM1+ macrophages, and a few eosinophils. CD20 B and CD56 NK cells were negative, as was granzyme B. Interestingly, we found ETCV discordance between the twins, but similar immune responses were observed in the twin, as the two chorionic vessels showed identical inflammatory infiltrate with the same immunophenotype. There was thus a predominance of Treg cells of a fetal origin, and this finding was correlated with a natural Treg response found in the fetal immune system [6]. However, functional studies on cytokines, such as IL-10 and TGF-β, may shed light on Tregs function in this kind of placental inflammatory process. In general, TGF-β regulates T-cell development and activity maintaining immunological homeostasis, especially during intrathymic differentiation [17]. IL-10 is a regulatory cytokine involved in innate and adaptive immune response. It plays a key role in modulating tissue inflammation and self-tolerance [18].

In our Di-Di placenta, another intriguing finding was TIA-1 expression. TIA-1, also known as T-cell intracellular antigen 1, is a constituent of cytotoxic T lymphocyte granules. It regulates transcription, pre-mRNA splicing, and mRNA stability and/or translation in many inflammatory conditions that involve cell signaling, metabolism, cell proliferation, angiogenesis, and cell death. Apoptosis induced by TIA-1 can be responsible for many adverse immune reactions such as organ transplant rejection, Crohn’s disease and ulcerative colitis, food allergies, aplastic anemia, and platelet impairment. On the other hand, TIA-1 expression has been found to be a prognostic marker in cancer immune response mediated by T-CD8 cytotoxic lymphocytes [19].

Although never reported in ETCV, TIA-1 expression can be found in other types of vasculitis, such as Kawasaki disease (KD), especially in medium-sized arteries, in which CD8+/TIA-1+ T cytotoxic lymphocytes are abundant [20] or in vasculitic neuropathy, with the same immunophenotypic pattern [21]. Of note, CD8+ T cells are involved in the antigen-dependent immune response and in antigen-independent immunity, after cytokine activation. Vasculitis is included in the latter condition, especially affecting small muscular arteries, such as polyarteritis nodosa (PAN), localized PAN, and rheumatoid vasculitis [20].

Analogously to other types of vasculitis, TIA-1 expression in ETCV should be subject to further in-depth analysis, especially on a larger case sample. Although few CD8+ T lymphocytes were TIA-1 positive, these cells may have a role in ETCV pathophysiology, potentially being involved in an antigen-dependent or independent response.

As previously mentioned, ETCV and VUE can often coexist [6]. In VUE, lymphocytes of maternal origin have been proved by human leukocyte antigen (HLA) mismatch and are considered a type of autoimmune response against the fetus [22,23,24,25]. Maternal T cells are chemotactically attracted into the villous stroma by activated fetal Hofbauer cells, being a unique allograft rejection [26,27]. Villous injury may be mediated by the perforin/granzyme pathway and C5b-9, which could lead to villous apoptosis [28]. In our Di-Di placenta, in the same territory of ETCV, multifocal areas of high-grade VUE were observed and associated with CHI. Immunophenotype of VUE displayed CD3+/CD25+ T lymphocytes, with CD4+ and CD8+ T subtypes in a similar ratio, and focal expression of TIA-1. Granzyme B, CD20 B lymphocytes, and CD56 natural killer cells were negative. According to the current literature, TIA-1 expression in VUE has never been reported. This finding may suggest an alternative way of villous damage, and, interestingly, the maternal immune response in VUE seems concordant with the fetal counterpart in ETCV.

CHI may represent an additional inflammatory response, being characterized by maternal histiocytes +/− lymphocytes within the intervillous space. It underlies an alloimmune cause, more rarely infective, such as malaria, cytomegalovirus, and SARS-CoV-2 [11,29]. CHI severity seems related to fetal growth restriction (FGR) and low birthweight [30], as well as VUE, which can also recur in subsequent pregnancies [31].

The combined effects of ETCV and VUE on fetal health are still uncertain. Meanwhile, the effects in stillbirth were reported in only one case [2]. In the small series described by Nohr and Wright [7], IUGR was present in one case, associated with ETCV and VUE. In our case, the placental territory of the female twin with SGA presented ETCV, VUE, and CHI. This combination might have affected fetal growth through impaired vascular and parenchymal exchange. Interestingly, we found TIA-1 expression in ETCV (fetal response) and in VUE (maternal response). These findings may suggest a common antigen or a common chemokine pathway to which both mother and fetus responded to. Further investigations are necessary, perhaps on a larger sample size and with long-term follow-up, in order to discover maternal and/or fetal hypersensitivity or autoimmune disorders.

## Figures and Tables

**Table 1:** Antibodies used to characterize the inflammatory response.

Antibody	Clone	Company
CD3	2GV6 Rabbit Monoclonal	Ventana Group, Milan, Italy
CD4	SP35 Rabbit Monoclonal	Ventana Group, Milan, Italy
CD8	SP57 Rabbit Monoclonal	Ventana Group, Milan, Italy
CD20	L26 Mouse Monoclonal	Ventana Group, Milan, Italy
CD25	4C9 Mouse Monoclonal	Cell Marque, Rocklin, CA, USA
CD68	PGM1 Mouse Monoclonal	DBS, Pleasanton, CA, USA
CD56	MRQ-42 Rabbit Monoclonal	Ventana Group, Milan, Italy
Granzyme B	Rabbit Polyclonal	Cell Marque, Rocklin, CA, USA
TIA-1	2G9A10F5 Mouse Monoclonal	Bio-Genex, Fremont, CA, USA

## Data Availability

The data presented in this study are available on request from the corresponding author.
